# Appendiceal Signet Ring Cell Carcinoma Presenting As Acute Appendicitis: A Case Report

**DOI:** 10.7759/cureus.59137

**Published:** 2024-04-27

**Authors:** Emily A Ina, Alexandria Sobczak, Konrad Drzymalski, Alejandro Biglione

**Affiliations:** 1 Osteopathic Medicine, Nova Southeastern University Dr. Kiran C. Patel College of Osteopathic Medicine, Fort Lauderdale, USA; 2 Internal Medicine, Wellington Regional Medical Center, Wellington, USA

**Keywords:** rare neoplasm, exploratory laparoscopy, gastrointestinal tumor, mimicker of acute appendicitis, appendiceal signet ring cell carcinoma

## Abstract

Appendiceal signet ring cell carcinoma is an exceedingly rare neoplasm which makes up only 4% of carcinomas of the appendix. It is a rare cause of abdominal pain which can mimic acute appendicitis. This case reports a 77-year-old female who presented to the emergency room with a complaint of right lower quadrant abdominal pain. After exploratory laparoscopy and histopathological studies, the patient was found to have peritoneal carcinomatosis and appendiceal signet ring cell carcinoma. This diagnosis unfortunately carries a relatively poor prognosis due to its aggressive nature. This study discusses the etiology, prevalence, clinical findings, and treatment of a rare cause of abdominal pain. This report sheds light on the importance of early detection and treatment of appendiceal signet ring cell carcinoma.

## Introduction

The incidence of primary appendiceal neoplasms is exceedingly low, accounting for less than 1% of all gastrointestinal tumors, and occurring in roughly 0.97 per 100,000 individuals [1]. Appendiceal signet ring cell carcinoma (ASRCC) is a rare subset of carcinoma of the appendix, making up only 4% of all cases [2]. Signet ring cell carcinoma (SRCC), most commonly, involves the stomach, followed by the colon and the lungs [3]. In this particular case, the appendix serves as an exceedingly unusual site for the development of signet ring cell carcinoma. Its clinical manifestation is subtle, often masquerading as other sources of acute abdominal distress, thereby complicating the diagnosis.

This study describes a 77-year-old female patient who presented to the emergency department with severe right lower quadrant abdominal pain originally believed to be due to acute appendicitis. Upon surgical exploration, ASRCC with metastasis was discovered. This report discusses the etiology, prevalence, clinical presentation, histologic characteristics, and therapeutic approaches for the management of ASRCC. Overall, the goal of reporting this case and continuing research is to emphasize the importance of considering ASRCC as a differential in patients presenting with nonspecific or atypical abdominal symptoms.

## Case presentation

A 77-year-old female with a medical history of hypertension, diabetes mellitus, and hyperthyroidism presented to the emergency department with a five-day history of waxing and waning right lower quadrant abdominal pain. She denied any associated nausea, vomiting, fever, and chills.

Upon admission, the patient appeared uncomfortable but awake, alert, and oriented to person, place, and time. She was afebrile with a heart rate of 95 beats per minute, respiratory rate of 20 breaths per minute, blood pressure of 152/88 mmHg, and oxygen saturation of 96% on room air. On physical examination, the cardiovascular examination was unremarkable, and the respiratory examination showed symmetric chest wall rise, lungs were clear to auscultation bilaterally and there was no evidence of accessory muscle use. Abdominal examination revealed a distended and tympanic abdomen with diffuse tenderness to palpation most prominent in the right lower quadrant, with associated positive bowel sounds. Further, there were no palpable abdominal masses or adenopathy appreciated.

**Figure 1:**
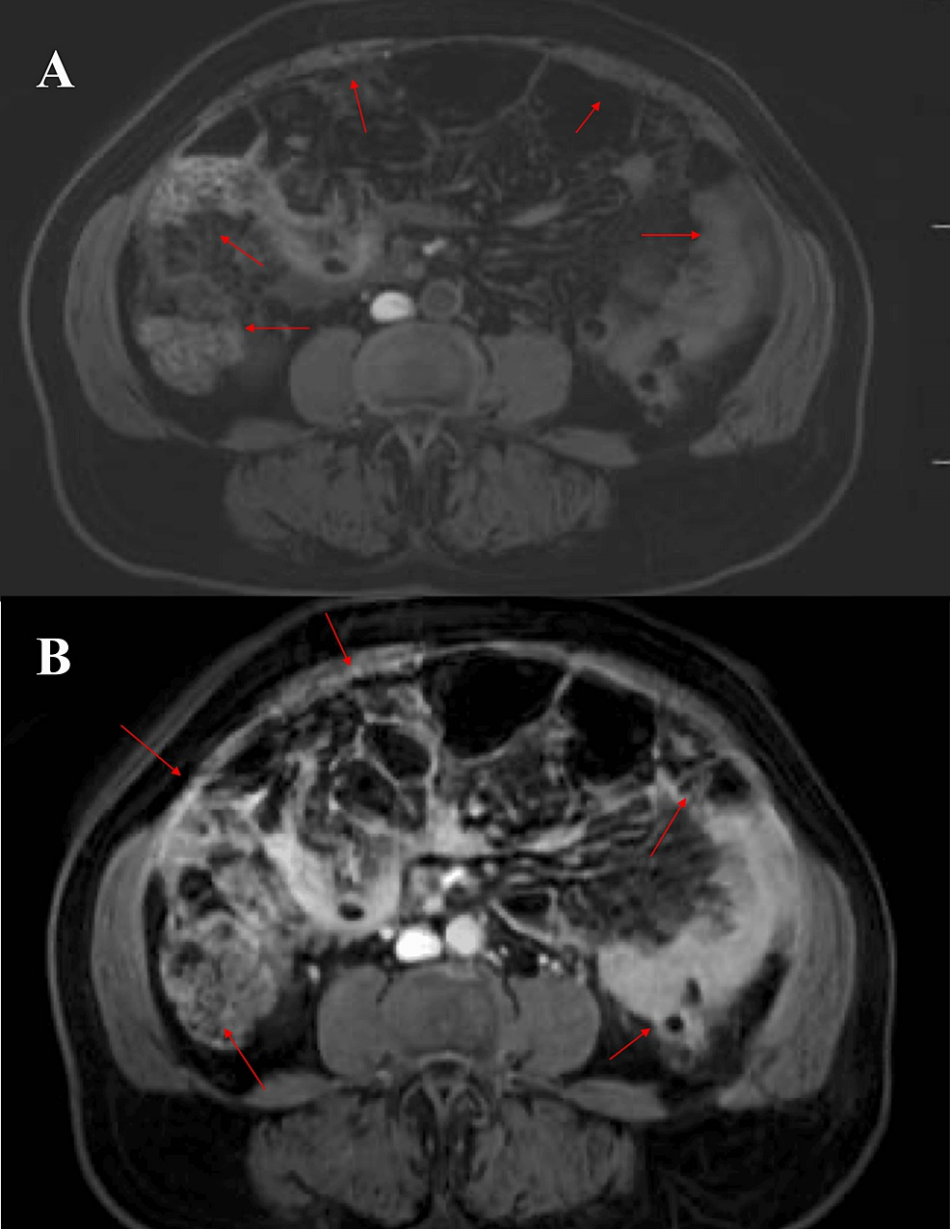
Abdominal T1-weighted axial view MRI without contrast (A) and T2-weighted axial view MRI with contrast (B). Appendiceal and ileocecal thickening with abdominal and pelvic ascites, peritoneal thickening, and omental nodularity (arrows).

**Figure 2:**
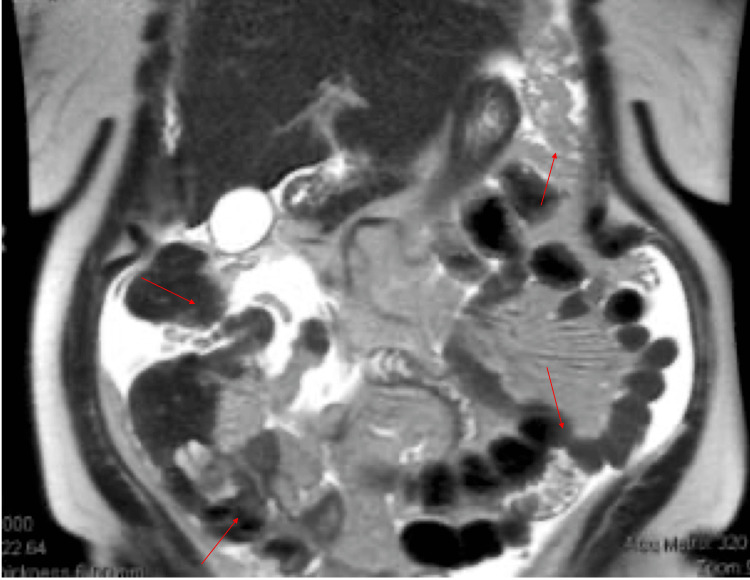
Abdominal T2-weighted coronal view image with contrast. Image demonstrating appendiceal and peritoneal thickening and nodularity concerning perforated appendicitis with peritonitis versus appendix malignancy with carcinomatosis (arrows).

The patient underwent further examination via radiological studies. Computed tomography (CT) of the abdomen displayed intraabdominal fluid with evidence of a dilated edematous appendix with fecalith concern for perforated appendicitis. The CT also noted a hyperdense lesion measuring 1.1 cm (~0.43 inches) within the hepatic parenchyma suggestive of a benign biliary hamartoma versus liver cysts. Additionally, a normal enhancing pancreatic parenchyma without pancreatic ductal dilation was noted and a right bladder-based diverticulum measuring 1.6 cm (~0.63 inches) was noted. Magnetic resonance imaging (MRI) of the abdomen, with and without contrast, further illustrated, in accordance with the radiology report, appendiceal and ileocecal thickening, a small amount of abdominal and pelvic ascites, peritoneal thickening, and omental nodularity. Further, it was reported that differential considerations should include perforated appendicitis with peritonitis versus appendix/ileocecal malignancy with carcinomatosis (Figures 1A, 1B, 2).

**Figure 3:**
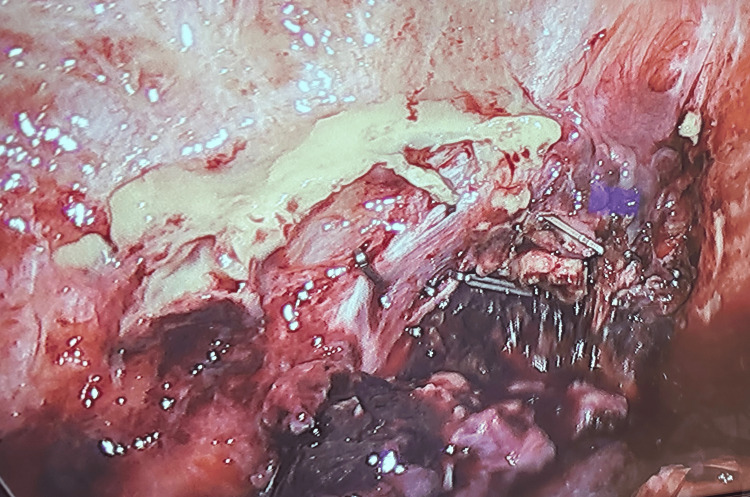
Intraoperative imaging of carcinomatosis of the abdominal wall.

The patient underwent a diagnostic laparoscopy with abdominal washout and drain placement. The patient tolerated the procedure well with no residual infection of the surgical sites and was promptly discharged. Findings during the laparoscopy included carcinomatosis of the abdominal wall and fungating 4-5 cm mass in the right lower quadrant (Figure 3). A total of three excisional biopsies of an abdominal wall mass measuring greater than 3 cm and less than 10 cm were obtained. Intraoperatively, the appendix was not visualized due to a highly inflamed area that was unsafe to proceed.

Histopathology studies revealed low-grade features of signet cell carcinoma in all three biopsies taken from the abdominal wall invading soft tissue and most likely the ovary, based on the presence of corpus albicans in the sample. Immunohistochemistry showed positive expression of CK20 and CDX2 and negative expression of neuroendocrine markers including, synaptophysin, chromogranin, and CD56, ruling out goblet cell carcinoid of the appendix. Further, mucicarmine highlighted the presence of intracellular mucin. With the advanced progression of peritoneal carcinomatosis, the patient may also be at increased risk for malnutrition, small bowel obstruction, and functional decline. Due to the rare nature of primary ovarian signet ring carcinomas, the patient was scheduled for a colonoscopy to assess for the possibility of colorectal origin and for the placement of a chemotherapy port with the recommendation of an outpatient positive emission tomography (PET) scan.

## Discussion

Primary appendiceal cancer itself is a rare diagnosis, accounting for less than one percent of cancer diagnoses each year in the United States [4]. Among appendiceal cancers, the following four different subtypes exist: mucinous adenocarcinoma, goblet cell carcinoids, intestinal-type adenocarcinomas, and signet ring cell carcinoma [5]. Primary appendiceal signet ring cell carcinoma remains the rarest subtype, constituting just 4% of all cases associated with carcinoma of the appendix [2]. Its aggressive, metastatic nature is usually indicative of a poor prognosis, with an 18% five-year survival rate [6]. Thus, early identification of ASRCC is of crucial importance to extend treatment options and to improve patient outcomes for this exceedingly rare diagnosis.

The clinical presentation of ASRCC is nonspecific, predominantly manifesting as lower abdominal pain. This serves as one of the primary reasons for the diagnostic challenges surrounding the identification of ASRCC, as the nonspecificity of presenting symptoms and the lack of pathognomonic signs can commonly overshadow underlying neoplasm with respective nonspecific radiographic findings further occluding preoperative diagnosis [2,7]. Because of the nonspecific presentation, rare occurrence, and aggressive nature, ASRCC metastases into adjacent structures within the abdominal cavity such as ovaries, pelvic lymph nodes, and peritoneal surface are commonly found at the time of diagnosis [7]. Signet ring cell carcinoma most commonly manifests within the gastrointestinal tract, with up to 90% of tumors arising from the stomach, colon, or breast [8].

As previously mentioned, ASRCC often lacks distinctive radiographic findings, further enhancing the difficulty of preoperative diagnosis. The initial suspicion of perforated appendicitis based on CT findings reflects the challenges in distinguishing ASRCC from other inflammatory conditions. While findings such as non-homogeneous masses in the appendiceal area may suggest the possibility of a malignant lesion on CT or MRI, diagnosis requires histopathological examination of biopsied tissue samples which may display signet ring cells containing abundant mucin within the cytoplasm and a displaced nucleus to the peripheral of the cell [8]. This, in turn, limits the possibility of a preoperative diagnosis until a postoperative examination may occur.

This case outlines the detrimental impact of ASRCC, as reflected by the carcinomatosis and metastatic spread visualized upon intraoperative examination. The ovaries, pelvic lymph nodes, and peritoneal surfaces remain the most common metastatic sites of signet ring cell carcinoma [9]. Thus, the decision to perform a colonoscopy for the assessment of a possible colorectal origin outlines the multidisciplinary approach required to manage such an aggressive and rare neoplasm. The presence of a disseminated disease process upon laparoscopic exploration reflects the challenges related to delayed diagnosis and the limitation of treatment options for a given patient with ASRCC. Furthermore, it was only upon immunohistochemical and pathological examination postoperatively, paired with the associated negative expression of synaptophysin, chromogranin, and CD56, that allowed for the exclusion of alternative diagnoses.

This case further presents the rarity of appendiceal manifestation of SRCC and the associated compounded difficulty in the differentiation of more common appendiceal pathologies as opposed to insidious carcinomas. The noted elusive and aggressive characteristics of ASRCC, accentuate the critical need for a comprehensive diagnostic assessment and the implementation of a multidisciplinary approach. This approach is paramount to achieving optimal patient outcomes and underscores the importance of nuanced clinical consideration in the context of rare and challenging neoplasms such as ASRCC.

## Conclusions

In conclusion, this study serves as an illustrative representation of the intricate diagnostic complexities entailed in primary ASRCC. Beyond its diagnostic implications, this case underscores the imperative significance of considering the occurrence of rare malignancies in individuals presenting with nonspecific symptoms. As more awareness is raised towards ASRCC, earlier detection can hopefully be established with the overall goal of improving the outcomes for patients with this aggressive malignancy.
